# The Effect of Target Speed and Verbal Instruction on NPC Measures in a Young, Healthy, and Active Population

**DOI:** 10.16910/jemr.12.4.5

**Published:** 2019-10-23

**Authors:** Ian McGinnis, Ryan Tierney, Jamie Mansell, Jacqueline Phillips

**Affiliations:** Temple University , Philadelphia, PA, USA

**Keywords:** Eye movement, convergence, attention, concussion, convergence insufficiency, assessment

## Abstract

**Purpose:** Evaluate the effect of target speed and verbal instruction on near point of convergence (NPC) measurements in a young, healthy, and active population. **Methods**: NPC was measured in 20 individuals with three target speeds and two sets of verbal instruction. The target speeds used were 1 cm/s, 3 cm/s, 5 cm/s, and participant self-paced. The verbal instruction given was either to indicate when the target became “double” or “blurry”. **Results**: Paired-samples t-tests revealed significant differences between 5 cm/s (5.44 ± 2.01) and 1 cm/s (6.72 ± 2.39, *p* = .003), 3 cm/s (6.10 ± 2.36, *p* = .030) and self-paced (6.63 ± 2.26, *p* = .005). A significant difference (*p* < .001) was also found between the “double” (6.72 ± 2.39) and “blurry” (10.82 ± 3.08) conditions. **Conclusion**: For young, healthy and active individuals, target speed and verbal instruction matter when measuring NPC.

## Introduction

A concussion can present clinically in a myriad of ways across a range of domains, including clinical symptoms, physical signs, cognitive impairment, neurobehavioral features, and sleep/wake disturbances [1]. Each year, an estimated 10,500 [2] and 135,000 [3] concussions occur at the NCAA and high school levels, respectively. Traditional assessment models recommend a focus on symptomology, cognition, and postural control [4, 5]. These traditional assessment techniques, however, do not adequately assess all of the domains known to be affected by concussion [6, 7]. Recently, authors have advocated for more comprehensive and multimodal assessment techniques, including vestibular and oculomotor assessments [1, 6-8]. One such oculomotor assessment that has shown promising diagnostic and prognostic value is the near point of convergence (NPC) [9-11]. 

NPC is a measure of the nearest point at which an individual’s eyes can maintain binocular focus on a target [12]. The measurement is performed by slowly moving a target linearly toward the bridge of the patient’s nose until they experience diplopia or ocular malalignment is observed by the examiner, at which point a measurement is taken. Previous authors have shown a significant difference between NPC measures in concussed and non-concussed individuals [9-11]. Additionally, this measurement has excellent reliability [13] and has been shown to increase diagnostic accuracy of a concussion assessment when combined with other tests [14]. An abnormal NPC is one of the main diagnostic criteria for convergence insufficiency (CI) [12, 15], which is a common sequela of sport-related concussion that occurs in 24-54.5% of patients [16-19] versus just 5% in the general population [15]. CI presents with symptoms similar to concussion, including headache, eye-strain, difficulty with reading, blurred vision, and diplopia [15, 20]. Moreover, post-traumatic CI has been associated with more severe overall symptomology and protracted recovery from concussion [10, 21-22]. 

 Despite proven clinical utility, there is considerable variation in how the NPC measurement is administered. While there are a number of target types used in the literature, it is largely agreed upon that the type of target used causes no clinically meaningful difference on NPC measures [23-25]. One aspect of NPC measurement that has yet to be investigated is target speed. Studies that use NPC distance as an outcome measure either do not report target speed [17, 19, 26-29] or report varying target speeds, ranging from 1 cm/s [30], 1-2 cm/s [23-25, 31-34], 3-5 cm/s [10], to patient self-paced [11, 13, 16, 18, 35]. Another variable that can potentially alter NPC measurement is when patients experience a failure of accommodation (i.e., blurred vision) before they experience diplopia. As the target approaches the patient’s face, the image becomes blurred, just before diplopia occurs. While this is a valid measurement known as accommodative amplitude (AA), clinicians administering the NPC are only interested in when double vision occurs. This discrepancy between “blurred” and “double” vision is one that has the potential to confuse the patient and cause them to report diplopia before it actually occurs, resulting in an inaccurate measurement.

 As researchers work to optimize concussion assessment tools for clinicians, it is crucial that consistent assessment techniques be used. The development of cut-off points (i.e., absolute or deviation from baseline) should be done with all aspects of administration and variance in mind. If target speed does have some influence on the outcome of NPC, assessments could generate false positive and/or false negative results, leading to inaccurate and suboptimal diagnoses and management of the injury. There are currently no studies that examine the effect of target speed or verbal instruction given to the patient on NPC measures. Therefore, the purpose of this study is to 1) evaluate the effect of target speed on NPC measures and 2) examine the difference within patients of when the target becomes “blurry” (AA) as opposed to when the target becomes “double” (NPC) in a young, healthy, and active population. 

## Materials & Methods

This study utilizes a repeated measures design. The independent variables are target speed (i.e., 1 cm/s, 3 cm/s, 5 cm/s, and patient self-paced) and verbal instruction (i.e., reporting of diplopia and blurriness). The dependent variable is near point of convergence (NPC) measures under the varying conditions. The Bernell Accommodation Convergence Ruler (Bernell, Mishawaka, IN) was used to assess NPC. The convergence ruler is 50 cm long and fitted with a sliding mechanism upon which a miniaturized Snellen eye chart is mounted. For NPC measurement, the participants were seated while maintaining upright posture with their head facing forward. The accommodative ruler was steadied upon the participant’s upper lip, just below their nose so that they could easily focus on the target in front of them. The target was a 12pt font letter on the Snellen eye chart. The target was moved slowly toward the participant’s face until they reported diplopia or until outward deviation of the eyes was observed by the examiner(s). 

**Figure 1:**
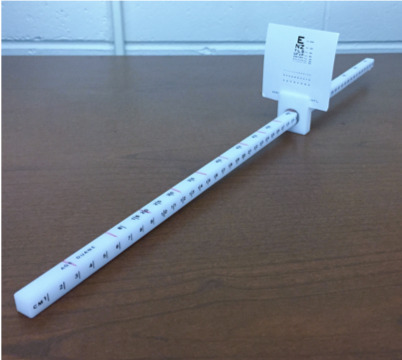
Bernell Accommodation Ruler with tick mark modification.

To assist in attaining the desired target speeds, a minor modification was made to the convergence ruler. The sliding mechanism on the convergence ruler was rotated such that the tick marks in 1 cm increments were facing to the right of the participant. On the newly designated top of the ruler, tick marks were drawn in red permanent marker in 3 cm increments from 0-30 cm (Figure 1). To attain the desired target speeds, the researcher played a metronome at varying beats per minute (BPM) while matching the beats with the movement of the sliding mechanism over newly drawn red tick marks on the ruler. To achieve a target speed of 1 cm/s, 3 cm/s, and 5 cm/s, the metronome was set to 20, 60, and 100 BPM respectively. 

**Figure 2:**
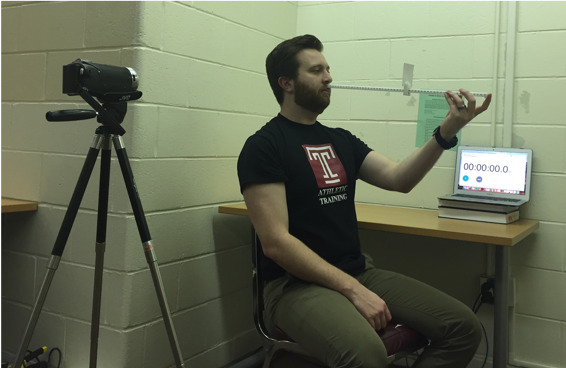
Participant positioning during data collection.

A digital camera mounted on a tri-pod was used to confirm accuracy in hitting the desired target speeds. The camera was set up to the right of the participant such that it could capture that tic marks on the ruler as well as the digital stopwatch in the background of the image (Figure 2). Video of the trials was reviewed after data collections so that target speeds could be calculated by reviewing the start and end-point of the target as well as the time elapsed during the trial. The self-paced trial was performed in a similar manner, except the target was moved at a self-determined pace that was comfortable for participant. These trials were also recorded so that target speed could be calculated. 

Testing took place over the course of two sessions scheduled within seven days of each other. Upon enrollment in the study, participants were randomly assigned their order of conditions to be tested. Each condition was tested for three trials with at least 30 s of rest between each trial. Three conditions (i.e., nine trials) were tested at the first session and two conditions (i.e., six trials) were tested at the second session, for a total of 15 trials. During data collection, a second examiner was present to observe the participant’s eyes for outward deviation as the first examiner was focused on maintaining accurate target speed. For the blurry condition, a target speed of 1 cm/s was used, and participants were specifically instructed to indicate to the examiner when the target became blurry at which point a measurement was taken. 

Data were analyzed using descriptive and inferential statistics with SPSS version 25 (IBM, North Castle, NY). A repeated measures ANOVA was performed to examine the effect of speeds (i.e., 1 cm/s, 3 cm/s, 5cm/s, and self-paced) on NPC measurements. Follow-up paired samples t-tests were performed using a Bonferonni adjusted alpha level of 0.008 (0.05/6). A separate paired samples t-test was used to examine the effect of verbal instruction (i.e., “double” versus “blurry”) on NPC measurements. 

## Participants

Twenty healthy and physically active university students, 10 male and 10 female, aged 24.3 ± 2.92 years were recruited for participation. Inclusion criteria consisted of being between the ages of 18 and 30 and exercising three times per week for 30 minutes or more at a time. Exclusionary factors were a history of brain injury within the past six months, uncorrectable or abnormal vision, and known ocular pathology. A health history questionnaire was used to determine participant eligibility. Participants answered a battery of questions regarding age, gender, weekly activity level, history of concussions, and ocular health. 

## Results

Descriptive statistics of NPC measures under the varying speed and verbal conditions are summarized in Table 1. A one-way repeated measures ANOVA revealed a significant difference among the target speed conditions, F(3,57) = 5.12, *p* - .003. Follow-up paired-samples t-tests revealed significant differences between 5 cm/s (5.44 ± 2.01) and 1 cm/s (6.72 ± 2.39, *p* = .003), 3 cm/s (6.10 ± 2.36, *p* = .030), and self-paced (6.63 ± 2.26, *p* = .005). There was a significant difference (p < .001) between the “double” (6.72 ± 2.36) and “blurry” 10.82 ± 3.08) conditions (Table 2).

**Table 1:** Descriptive data for NPC

Speed	N	Mean	SD	Min	Max
1 cm/s	20	6.72	2.39	2.67	13.67
3 cm/s	20	6.10	2.36	2.83	11.67
5 cm/s	20	5.44	2.01	2.67	11.00
Self-paced	20	6.63	2.26	2.67	11.33
1 cm/s "blurry"	20	10.82	3.08	6.67	20.33
Note. cm/s = centimeters / second, SD = Standard Deviation, Min = Minimum, Max = Maximum					

**Table 2:** Paired-sample t tests for conditions

Pair	t	df	*p*
1 cm/s - 3 cm/s	1.69	19	.108
1 cm/s - 5 cm/s	3.47	19	.003*
1 cm/s - self-paced	0.257	19	.80
3 cm/s - 5 cm/s	2.30	19	.03*
3 cm/s - self-paced	-1.24	19	.23
5 cm/s - self paced	-3.19	19	.005*
1 cm/s - 1 cm/s "blurry"	-5.89	19	<.001*
Note. cm/s = centimeters / second, df = Degress of Freedom, * denotes significance (p ≤ 0.05)					

## Discussion

The results of this study showed that target speed and verbal instruction do measurably affect the outcome of an NPC assessment. The 5 cm/s condition resulted in a significantly shorter NPC distance than all other speed conditions including the self-paced trials.

As speeds increased, the NPC measurement tended to decrease. Additionally, verbal instruction (i.e. identifying when the target was “double” as opposed to “blurry”) yielded a significant difference in NPC distance.

The reason for this difference among target speeds is not clear, but it is likely not a physiological restraint as the eyes are capable of working at much greater speeds than 5 cm/sec [36]. We propose the following theoretical explanation for the difference. When the NPC assessment is administered by a clinician, a series of recognition and communicative cues need to take place to obtain an accurate measurement. While the clinician is moving the target toward the participant, the participant has to identify when the target has become “double.” Once they identify that the target is “double,” they have to communicate that fact to the clinician who then has to recognize that communication and stop moving the target. There is an inherent communication lag present in this process that may not be quantifiable in lower target speeds, but more noticeable in higher target speeds. Although the communication lag might last the same amount of time regardless of target speed, the distance traveled by the target during that time period is much greater at 5 cm/s versus 1 cm/s potentially resulting in the differences observed by the study.

The clinical and research implications of the present result are that target speed and verbal instruction matter. While it is likely not necessary to precisely measure the target speed being used in each assessment, making sure to not move too quickly and staying consistent are keys. According to our findings, as long as researchers and clinicians stay within the range of 1-3 cm/s, they should not see any inconsistencies due to target speed when examining a healthy population. An alternative to having a clinician administer the test could be to allow the patient to move the target at a speed that is comfortable to them as the mean target speed of the self-paced trials was 2.8 cm/s and the NPC measures were virtually identical to the 1 cm/s condition (6.63 cm versus 6.72 cm respectively).

Clinicians and researchers should take the time to provide explicit instructions before administering the test. When our participants were told specifically to report when the target became “blurry” the distance was significantly greater than when they were told to report the target becoming “double”. While AA is a valid ophthalmological measurement, it is not the measure of interest when assessing NPC. There are three actions occurring at the eyes when an individual is tracking an object from far to near known as the near triad: 1) medial rotation of the eyes caused by contraction of the medial recuts muscles, 2) the curvature of the eye’s lens is changes via contraction of the ciliary muscles to increase refractive power (i.e., accommodation), and 3) the pupil is constricted to increase the depth of field within the eye [37]. AA represents only a failure of accommodation and is not the equivalent to NPC. Ensuring that the patient knows their role in the assessment (i.e. identifying when the target is “double” and not “blurry”) is key to obtaining an accurate measurement.

Previous literature examining NPC has been largely homogenous in target speed, primarily ranging from 1-2 cm/s to examine a myriad of independent variables. Some studies fail to report target speed at all [17, 19, 26-29], but only one study [10] fell outside of the previously described range of 1-2 cm/s. DuPrey et al. (2017) used a target speed of 3-5 cm/s on a concussed population to examine if CI, as identified by a receded NPC resulted in prolonged recovery from concussion. The group concluded that individuals with post-traumatic CI were at a risk of prolonged recovery from concussion. Based on the results of the present study, it is unlikely that their choice of target speed would have influenced their outcome. One reason is that with a faster target speed, one would expect to see lower NPC values based on the present result. Secondly, so long as consistent measures were used for all subjects and the patients were able to be placed into distinct groups based solely on their NPC measures. The one clear problem with using this higher target speeds is the inability to compare the result to other literature. In order to make larger assertions about assessments like the NPC, consistent methods need to be used across the literature. 

Excluding the 5 cm/s condition, the average NPC for all trials and speeds from the present study was 6.48 cm. This figure is consistent with previous literature which examined NPC in healthy populations of a similar age using a reported target speed of 1-2 cm/s [23, 31, 34]. Studies using a participant self-paced target speed have been primarily on healthy children, but have found NPC measurements ranging from 1.9 - 3.3 cm [11, 13, 16]. The discrepancy between these figures and our average of 6.63 cm during the self-paced condition could be explained by individual differences or by other administrative variables. In the present study, NPC was measured from the target at patient reported diplopia to the participant’s upper lip. For each of the studies mentioned above using the self-paced method, NPC was measured as the distance from the target to the tip of the participant’s nose - this could perhaps be to blame for the minor difference between our result and theirs.

Slight differences in NPC have been found between target speeds when examining concussed individuals. Using a self-paced speed, Howell et al. (2017), Pearce et al. (2015), and Mucha et al. (2014) found NPC measures of 7.25 cm, 6.23 cm, and 5.9 cm respectively. Using a target speed of 3-5 cm/s, DuPrey et al. (2017) found an average NPC of 8.23 in their concussed sample. Like the aforementioned studies on healthy individuals, these minor differences could be attributed to a similar administrative variable. Howell et al. (2017), Pearce et al. (2015), and Mucha et al. (2014) all measured NPC as the distance from the target to the tip of the participant’s nose. DuPrey et al. (2017) measured from the target to the bridge of the participant’s nose. There are likely other variables at play including individual differences and, especially in the concussed population, severity of injury.

There are two primary limitations that both exist within the participant sample of the present study. The participants in this study were from a sample of convenience, and many were certified athletic trainers who were familiar with the administration of the NPC before taking part in this study. Their familiarity may have introduced some bias into the data in an unpredictable direction. The present sample also consisted entirely of healthy and physically active individuals. Future research on the topic should look to replicate the study on concussed individuals to see if they respond in a similar way to changes in target speed. Target speed and verbal instruction used during the administration of an NPC assessment can significantly influence the result. Target speeds ranging from 1-3 cm/s, and even self-paced seem to be consistent. However, a speed of 5 cm/s may result in shorter NPC distances leading to false negatives and ultimately suboptimal injury management. Ensuring that a patient understands their role in the assessment (i.e. identifying when the target is “double” and not “blurry”) is also imperative for obtaining an accurate measurement. If a patient misunderstands the directions and identifies when the target becomes “blurry” instead of “double”, the measurement will likely be much larger than expected and potentially generate false positive results. 

### Ethics and Conflict of Interest

The author(s) declare(s) that the contents of the article are in agreement with the ethics described in http://biblio.unibe.ch/portale/elibrary/BOP/jemr/ethics.html and that there is no conflict of interest regarding the publication of this paper.
